# *Mycobacterium marinum* Tenosynovitis following Steroid Injection in an Avid Seaman from Long Island, New York

**DOI:** 10.4269/ajtmh.19-0336

**Published:** 2019-12

**Authors:** Eric Sin, George Psevdos, Luis A. Marcos

**Affiliations:** 1 Department of Medicine (Division of Infectious Diseases), Stony Brook University, Stony Brook, New York;; 2 Northport Veterans Affairs Medical Center, Northport, New York;; 3 Department of Microbiology and Immunology, Stony Brook University, Stony Brook, New York

A 51-year-old man, resident of Long Island, New York, and an avid seaman (spends all the weekends on keeping his boat in a good sailing condition), presented to the emergency department with a nonhealing swelling of the left fourth finger. He had sustained an injury due to a wood splinter on the same finger while working on his boat about 15 months before admission. After removing the splinter, a painless chronic mild swelling persisted for months. A month before admission, he received in the 4th finger a local steroid injection for possible gout, which resulted in worsening of the swelling to the point that his wedding ring had to be cut off to alleviate the pressure.

A well-demarcated swelling and redness was noted at the left 4th proximal interphalangeal (PIP) joint and dorsum of the finger (Figure 1A). A magnetic resonance imaging showed increased T2-weighted signal and enhancement involving only the soft tissues and small amount of fluid on the 4th PIP joint and extensor tendons (tenosynovitis), but no enhancement of the bone (Figure 1B). His white blood cell count was 9,710 ells/cm^3^ and C-reactive protein was 1.1 mg/L. There was no history of immunosuppression; he refused an HIV test. An incision and drainage (I&D) was performed, but no gross purulence was found, except for an extensive granulation tissue down to the tendon sheath. The histology depicted necrotic debris and granulation tissue with many acid-fast staining organisms, and *Mycobacterium marinum* was isolated in AFB culture incubated at 32°C at day 10 from I&D (Figure 2A and B). The final diagnosis was tenosynovitis due to *M. marinum*.

**Figure 1.**
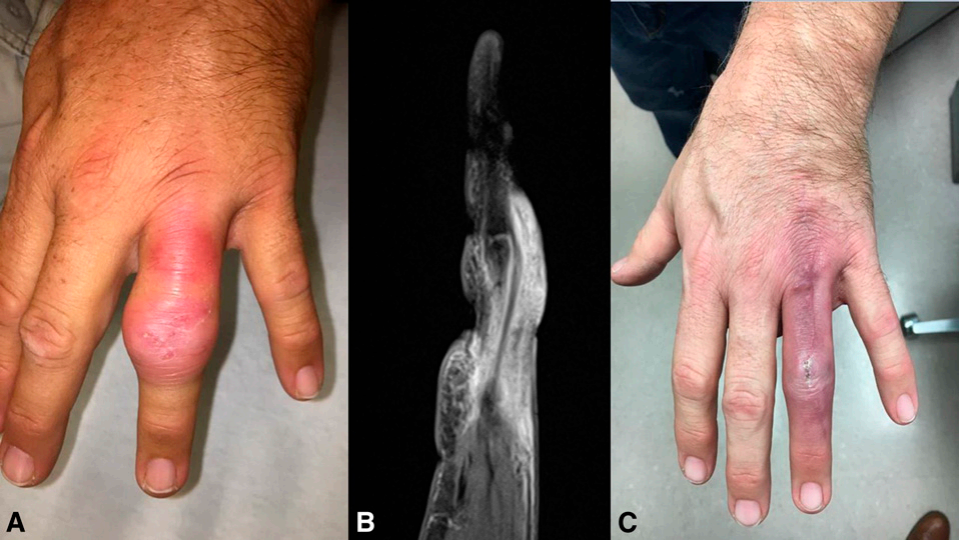
(**A**) Tenosynovitis of the ring finger. (**B**) Magnetic resonance imaging of the finger. (**C**) Appearance of the finger after 3 months of antibiotic treatment. This figure appears in color at www.ajtmh.org.

**Figure 2.**
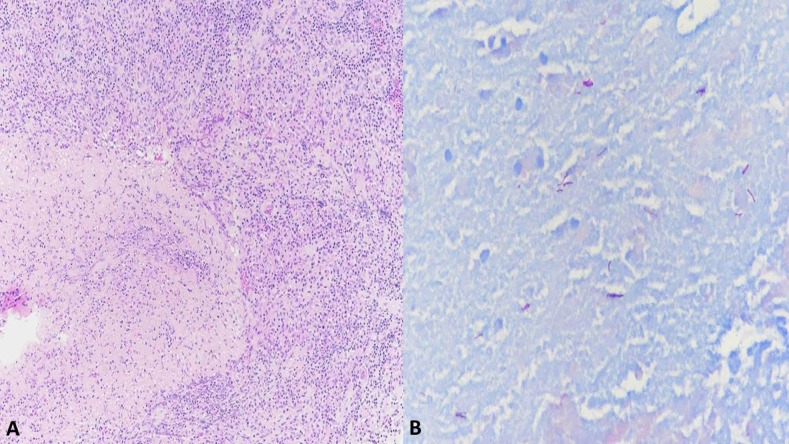
(**A**) Hematoxylin and eosin stain (magnification ×10): granulation reaction surrounding necrotic debris. (**B** ) Acid-fast stain showing many mycobacterial organisms (magnification ×40). This figure appears in color at www.ajtmh.org.

This is a fast-growing nontuberculous *mycobacterium* that is often acquired via traumatic injuries leading to infection by direct inoculation from fish fins, fish bites, or water from fish tanks.^1^ Clinical manifestations include papules, nodules, erythematous plaques, ulcerations, and sporotrichoid-like lesions mostly in the upper limbs. Cases of solitary nontender nodules on the hands of people working on boats have been reported.^2,3^

A failure to elicit the history of potential exposure and microbiologic features of this organism is responsible for the often-delayed diagnosis. The incubation period of *M. marinum* can be from weeks to months after the inoculation.^3^ Invasive form of the infection involves deep structures such as tendon, synovium, and bone; these infections are often identified following corticosteroid therapy. Our patient did receive a corticosteroid injection locally followed by clinical worsening, which may have contributed by both suppressing the immune system and inoculating the organism. Corticosteroids may decrease the cytokine profile of macrophages, leading to a reduced host defense against mycobacterial infections and granuloma formation,^4^ and they may inhibit the necrotic cell death of infected *Mycobacterium* cells, which may perpetuate the survival of the bacteria in the cells.^5^

In a recent meta-analysis, local or systemic suppression of the immune system by corticosteroids was a common culprit for invasive *M. marinum* infections.^6^ A delayed diagnosis was also observed (∼17 months from symptom onset).^6^

Histopathologic findings can range from nonspecific inflammatory infiltrate to poorly formed, well-formed, necrotizing, and non-necrotizing granulomas. Acid-fast staining organisms are most frequently localized in necrotic and suppurative cores of the granulomas.^7^

There is no established therapy of choice for *M. marinum* infections. Most cases of invasive infections require surgical debridement, and combination antibiotic treatment is needed, with duration of therapy ranging from 3 to 6 months depending on the degree of cutaneous involvement.^8^ Clarithromycin with rifampin or ethambutol is the usual choice. Other antibiotics such as tetracyclines and trimethoprim–sulfamethoxazole can also be used.^8,9^ Our patient was treated with clarithromycin and rifampin for 12 months. No antibiotic susceptibility was performed by the laboratory of reference. Figure 1B depicts the finger after 3 months of antibiotics, and decision was made to extend therapy because of deep structure infection and reported failures with short regimens.^9^ At the end of the treatment, there was no more erythema or swelling, or pain with movement, and the incision was well healed.

## References

[1] Cheung JPY Fung B Wong SSY Ip WY, 2010 Review article: *Mycobacterium marinum* infection of the hand and wrist. J Orthop Surg 18: 98–103. 10.1177/230949901001800122 20427845

[2] Bryef-Groff BM Wilson BB, 2009 *Mycobacterium marinum* infection after a boating accident. Consultant Pediatricians: 8 Available at: https://www.pediatricsconsultantlive.com/pediatric-skin-diseases/mycobacterium-marinum-infection-after-boating-accident.

[3] Johnson RP Xia Y Cho S Burroughs RF Krivda SJ, 2007 Mycobacterium marinum infection: a case report and review of the literature. Cutis 79: 33–364. 17330619

[4] Rock RB Hu S Gekker G Sheng WS May B Kapur V Peterson PK, 2005 *Mycobacterium tuberculosis*-induced cytokine and chemokine expression by human microglia and astrocytes: effects of dexamethasone. J Infect Dis 192: 2054–2058. 16288367 10.1086/498165

[5] Gräb J 2019 Corticosteroids inhibit *Mycobacterium tuberculosis*-induced necrotic host cell death by abrogating mitochondrial membrane permeability transition. Nat Commun 10: 688. 30737374 10.1038/s41467-019-08405-9 PMC6368550

[6] Lahey T, 2003 Invasive *Mycobacterium marinum* infections. Emerg Infect Dis 9: 1496–1498. 14725262 10.3201/eid0911.030192 PMC3035536

[7] Sia YT 2016 Clinical and pathological evaluation of *Mycobacterium marinum* group skin infections associated with fish markets in New York city. Clin Infect Dis 62: 590–595. 26673347 10.1093/cid/civ937

[8] Franco-Paredes C Marcos LA Henao-Martinez AF Rodriguez-Morales AJ Villamil-Gomez WE Bonifaz A, 2019 Cutaneous Mycobacterial infections. Clin Microbiol Rev 32: e00069–18. 10.1128/CMR.00069-18 PMC6302357 30429139

[9] Aubry A Chosidow O Caumes E Robert J Cambau E, 2002 Sixty-three cases of *Mycobacterium marinum* infection: clinical features, treatment, and antibiotic susceptibility of causative isolates. Arch Intern Med 162: 1746–1752. 12153378 10.1001/archinte.162.15.1746

